# Supplementary material for: Inter-Device Reliability of a Three-Dimensional Markerless Motion Capture System Quantifying Elementary Movement Patterns in Humans
Source: J Funct Morphol Kinesiol. 2023 May 22;8(2):69. doi: 10.3390/jfmk8020069 (PMC10204374; doi:10.3390/jfmk8020069)
Supplement: Supplementary file 1 [file jfmk-08-00069-s001.zip › jfmk-2374135-supplementary.pdf]

Table S1. Comparison data for inter-device reliability (technical reliability) for two identical markerless motion capture systems. Results are presented as x̄±SD for normally distributed data, or medians for non-normally distributed data. Significance (*p*-value) for individual *t*-tests mean absolute differences (x̄-diff), effect sizes (ES), and intra-class correlation coefficients (ICC) are also listed for each variable.

Anatomical Motion	System 1	System 2	*p*-value	x̄-diff	ES	ICC
Shoulder Abduction						
Shoulder abduction mobility, maximum left value (°)†	179.3	178.3	0.82	0.6	0.38	0.89
Shoulder abduction mobility, maximum right value (°)†	178.5	177.1	0.86	0.6	0.06	0.94
Shoulder Horizontal Abduction						
Shoulder horizontal abduction mobility, maximum left value (°)†	37.1	34.4	0.21	3.3	0.41	0.91
Shoulder horizontal abduction mobility, maximum right value (°)	44.6 ± 19.5	42.3 ± 16.1	0.68	2.4	0.13	0.91
Shoulder Internal/External Rotation						
Shoulder external rotation, maximum left value (°)	84.3 ± 11.0	85.4 ± 12.3	0.76	1.1	0.10	0.95
Shoulder external rotation, maximum right value (°)	92.7 ± 10.0	92.4 ± 9.8	0.93	0.3	0.03	0.93
Shoulder internal rotation, maximum left value (°)	70.1 ± 18.8	70.3 ± 18.1	0.98	0.1	0.01	0.98
Shoulder internal rotation, maximum right value (°)	68.5 ± 16.4	69.5 ± 15.3	0.84	1.0	0.06	0.95
Shoulder Flexion/Extension						
Shoulder flexion, maximum left value (°)†	182.5	182.7	0.76	1.6	0.10	0.34
Shoulder flexion, maximum right value (°)†	183.6	186.5	0.25	3.6	0.37	0.50
Shoulder extension, maximum left value (°)†	53.4	54.3	0.84	0.9	0.07	0.68
Shoulder extension, maximum right value (°)	54.4 ± 16.6	54.1 ± 15.5	0.96	0.3	0.02	0.98
Forward Fold						
Forward fold - thoracic flexion, maximum value (°)†	33.1	34.1	0.90	1.0	0.04	0.83
Forward fold - lumbar flexion, maximum value (°)†	55.6	57.8	0.74	0.4	0.11	0.85
Trunk Rotation						
Thoracic rotation, maximum left value (°)	34.8 ± 7.7	33.6 ± 8.0	0.64	1.2	0.15	0.75
Thoracic rotation, maximum right value (°)†	29.8	28.3	0.60	0.9	0.17	0.81

Lumbar rotation, maximum left value (°)	13.5 ± 3.7	13.5 ± 3.8	0.99	<0.1	0.01	0.80
Lumbar rotation, maximum right value (°)	14.9 ± 3.6	14.0 ± 3.4	0.41	0.9	0.26	0.69
Trunk Lateral Flexion						
Thoracic lateral flexion, maximum left value (°)	30.3 ± 6.2	28.4 ± 5.8	0.34	1.9	0.31	0.84
Thoracic lateral flexion, maximum right value (°)†	30.5	25.9	0.04*	3.3	0.70	0.68
Lumbar lateral flexion, maximum left value (°)	15.4 ± 3.0	14.1 ± 3.1	0.18	1.3	0.43	0.78
Lumbar lateral flexion, maximum right value (°)†	15.7	13.0	0.03*	2.2	0.74	0.57
Reverse Lunge with Rotation						
Thoracic rotation, maximum left value (°)	18.6 ± 12.2	23.6 ± 11.9	0.19	5.0	0.42	0.59
Thoracic rotation, maximum right value (°)	13.7 ± 10.3	20.3 ± 10.0	0.05	6.6	0.65	0.47
Lumbar rotation, maximum left value (°)	8.8 ± 5.9	10.6 ± 4.6	0.29	1.8	0.34	0.78
Lumbar rotation, maximum right value (°) †	10.9	11.6	0.34	1.0	0.31	0.29
Thoracic flexion, maximum left value (°)	14.0 ± 6.6	12.1 ± 5.4	0.32	1.9	0.32	0.64
Thoracic flexion, maximum right value (°)	14.4 ± 6.7	13.2 ± 5.5	0.56	1.1	0.19	0.80
Lumbar flexion, maximum left value (°)	20.4 ± 9.9	18.1 ± 8.8	0.44	2.3	0.25	0.67
Lumbar flexion, maximum right value (°)	20.0 ± 9.6	19.3 ± 9.1	0.81	0.7	0.09	0.80
Standing Hip Abduction						
Hip abduction, maximum left value (°)	48.6 ± 9.4	50.6 ±11.1	0.56	1.9	0.19	0.84
Hip abduction, maximum right value (°)	52.6 ± 8.1	51.5 ± 9.4	0.69	1.1	0.13	0.74
Bilateral Squat						
Squat COM depth value (cm)†	50.6	51.2	0.80	0.4	0.09	0.96
Hip flexion, maximum left value (°)	94.6 ± 14.5	105.0 ± 14.0	0.03*	10.4	0.73	0.55
Lower extremity flexion, sum left value (°)	263.3 ± 25.7	269.0 ± 21.3	0.46	5.6	0.24	0.79
Lower extremity flexion, sum right value (°)	265.4 ± 26.5	263.2 ± 22.7	0.78	2.2	0.09	0.79
Trunk flexion value (°)	45.1 ± 8.7	44.3 ± 8.6	0.79	0.7	0.08	0.96
Hip adduction, left value (°)	3.84±3.57	4.39±3.28	0.61	0.6	0.16	0.75
Hip adduction, right value (°)	2.82±4.32	2.95±4.73	0.93	0.1	0.03	0.93
Dynamic valgus, left value (°)	6.21±4.93	5.06±3.27	0.39	1.2	0.28	0.47

Dynamic valgus, right value (°)	7.80±6.84	6.54±4.22	0.49	1.3	0.22	0.61
Overhead Squat						
Overhead squat COM depth value (cm)	48.7 ± 8.3	50.6 ± 9.6	0.51	1.9	0.21	0.92
Overhead squat hip flexion, maximum left value (°)†	108.7	115.4	0.38	5.9	0.29	0.82
Overhead squat hip flexion, maximum right value (°)†	104.1	107.8	0.28	6.0	0.36	0.51
Overhead squat knee flexion, maximum left value (°)	128.4 ± 13.4	128.5 ± 14.5	0.97	0.2	0.01	0.96
Overhead squat knee flexion, maximum right value (°)	129.3 ± 11.8	128.1 ± 14.6	0.78	1.2	0.09	0.82
Overhead squat ankle flexion, maximum left value (°)	39.6 ± 10.0	37.1 ± 8.6	0.40	2.5	0.27	0.72
Overhead squat ankle flexion, maximum right value (°)	40.6 ± 9.9	37.7 ± 7.7	0.30	2.9	0.33	0.54
Overhead squat lower extremity flexion, sum left value (°)	273.6 ± 29.3	275.7 ± 35.7	0.84	2.1	0.06	0.92
Overhead squat lower extremity flexion, sum right value (°)	267.2 ± 24.7	270.1 ± 34.0	0.76	2.9	0.10	0.80
Overhead squat, trunk flexion value (°)	39.7 ± 7.0	39.2 ± 6.9	0.83	0.5	0.07	0.86
Overhead squat hip adduction, left value (°)	4.76 ± 4.18	4.92 ± 3.64	0.90	0.2	0.04	0.89
Overhead squat hip adduction, right value (°)	3.82 ± 3.48	3.75 ± 4.04	0.96	0.1	0.02	0.74
Overhead squat dynamic valgus, left value (°)	6.26 ± 4.87	5.56 ± 2.95	0.58	0.7	0.17	0.56
Overhead squat dynamic valgus, right value (°)	6.48 ± 2.71	6.54 ± 3.94	0.95	0.1	0.02	0.64
Unilateral Squat						
Unilateral squat COM depth, left value (cm)	30.5 ± 9.1	30.2 ± 8.7	0.92	0.3	0.03	0.98
Unilateral squat COM depth, right value (cm)†	34.3	32.6	0.95	0.3	0.03	0.96
Unilateral squat hip flexion, maximum left value (°)†	75.2	85.0	0.22	8.5	0.40	0.81
Unilateral squat hip flexion, maximum right value (°)†	76.4	84.4	0.17	8.5	0.46	0.63
Unilateral squat knee flexion, maximum left value (°)	101.3 ± 15.4	100.0 ± 14.4	0.78	1.4	0.09	0.91
Unilateral squat knee flexion, maximum right value (°)	106.3 ± 13.4	104.3 ± 14.1	0.65	2.0	0.15	0.90
Unilateral squat ankle flexion, maximum left value (°)	39.1 ± 10.8	38.0 ± 8.3	0.74	1.0	0.11	0.75
Unilateral squat ankle flexion, maximum right value (°)	40.2 ± 10.6	36.8 ± 9.1	0.28	3.4	0.35	0.43
Unilateral squat lower extremity flexion, sum left value (°)	210.9 ± 35.8	217.8 ± 31.3	0.52	7.0	0.21	0.89
Unilateral squat lower extremity flexion, sum right value (°)	219.3 ± 32.1	222.5 ± 32.9	0.76	3.2	0.10	0.80
Unilateral squat lateral trunk flexion, left value (°)	7.876 ± 6.68	8.14 ± 4.74	0.89	0.3	0.05	0.71

Unilateral squat lateral trunk flexion, right value (°)	4.503 ± 6.39	5.27 ± 5.55	0.69	0.8	0.13	0.88
Unilateral squat pelvic obliquity, left value (°)	0.995 ± 8.74	2.25 ± 6.70	0.61	1.3	0.16	0.33
Unilateral squat pelvic obliquity, right value (°)	4.0 ± 15.1	2.5 ± 10.8	0.72	1.5	0.11	0.84
Unilateral squat hip adduction, left value (°)	18.8 ± 7.8	15.5 ± 6.4	0.14	3.4	0.47	0.75
Unilateral squat hip adduction, right value (°)	21.1 ± 8.5	21.1 ± 6.9	0.99	0.1	<0.01	0.64
Unilateral squat dynamic valgus, left value (°)	25.9 ± 12.8	33.5 ± 14.4	0.09	7.5	0.55	0.82
Unilateral squat dynamic valgus, right value (°)	24.5 ± 18.8	21.7 ± 13.7	0.59	2.8	0.17	0.38
Forward Lunge						
Forward lunge stride length, left value (cm)†	78.5	81.4	0.28	2.3	0.36	0.94
Forward lunge stride length, right value (cm)	81.5 ± 10.8	83.3 ± 11.4	0.61	1.8	0.16	0.97
Forward lunge trail hip extension, left value (°)	38.5 ± 17.8	37.0 ± 19.1	0.80	1.5	0.08	0.84
Forward lunge trail hip extension, right value (°)	44.5 ± 17.7	42.7 ± 19.1	0.76	1.8	0.10	0.89
Forward lunge hip flexion, maximum left value (°)	78.1 ± 14.2	77.8 ± 17.6	0.95	0.3	0.02	0.86
Forward lunge hip flexion, maximum right value (°)†	77.0	77.4	0.74	1.1	0.11	0.77
Forward lunge knee flexion, maximum left value (°)†	117.3	115.3	0.28	1.5	0.36	0.89
Forward lunge knee flexion, maximum right value (°)	116.8 ± 9.4	116.9 ± 7.8	0.98	0.1	0.01	0.92
Forward lunge ankle flexion, maximum left value (°)	26.4 ± 8.7	26.0 ± 7.1	0.86	0.4	0.06	0.79
Forward lunge ankle flexion, maximum right value (°)†	27.6	25.4	0.70	1.1	0.13	0.84
Forward lunge lower extremity flexion, sum left value (°)	220.4 ± 20.3	217.7 ± 20.3	0.68	2.7	0.13	0.86
Forward lunge lower extremity flexion, sum right value (°)†	226.3	222.6	0.68	1.7	0.14	0.82
Forward lunge lateral trunk flexion, left value (°)	1.6 ± 6.2	1.1 ± 6.3	0.80	0.5	0.08	0.93
Forward lunge lateral trunk flexion, right value (°)	2.3 ± 5.1	1.6 ± 5.0	0.68	0.7	0.13	0.68
Forward lunge pelvic obliquity, left value (°)	3.8 ± 10.7	0.2 ± 10.3	0.24	4.0	0.38	0.79
Forward lunge pelvic obliquity, right value (°)	3.9 ± 9.4	3.5 ± 7.9	0.88	0.4	0.05	0.44
Forward lunge hip adduction, left value (°)	10.9 ± 4.9	9.8 ± 6.1	0.55	1.1	0.19	0.80
Forward Lunge						
Forward lunge hip adduction, right value (°)	16.9 ± 7.6	14.7 ± 7.4	0.38	2.1	0.28	0.82
Forward lunge dynamic valgus, left value (°)	20.5 ± 13.6	15.8 ± 13.1	0.28	4.7	0.35	0.85

Forward lunge dynamic valgus, right value (°)	29.8 ± 14.9	26.7 ± 13.4	0.47	3.2	0.22	0.82
Lateral Lunge						
Lateral lunge stride length, left value (cm)	99.4 ± 10.3	99.6 ± 10.6	0.96	0.2	0.02	0.99
Lateral lunge stride length, right value (cm)†	99.6	98.1	0.57	1.7	0.19	0.73
Lateral lunge trail hip abduction, left value (°)	42.5 ± 6.5	43.3 ± 9.1	0.76	0.8	0.10	0.80
Lateral lunge trail hip abduction, right value (°)	40.1 ± 10.3	44.3 ± 7.3	0.14	4.2	0.47	0.54
Lateral lunge knee flexion, maximum left value (°)†	114.5	114.0	0.66	1.9	0.15	0.97
Lateral lunge knee flexion, maximum right value (°)†	120.0	113.2	0.31	4.4	0.33	0.87
Lateral lunge ankle flexion, maximum left value (°)	34.7 ± 11.3	33.8 ± 10.2	0.79	0.9	0.08	0.88
Lateral lunge ankle flexion, maximum right value (°)	39.4 ± 13.5	36.3 ± 10.8	0.43	3.1	0.25	0.66
Lateral lunge lower extremity flexion, sum left value (°)†	250.6	236.9	1.00	<0.1	<0.01	0.93
Lateral lunge lower extremity flexion, sum right value (°)	250.3 ± 37.0	246.4 ± 36.2	0.74	3.9	0.11	0.92
Lateral lunge hip flexion, maximum left value (°)	91.4 ± 15.1	95.3 ± 15.9	0.43	4.0	0.26	0.74
Lateral lunge hip flexion, maximum right value (°)	90.5 ± 18.0	93.9 ± 18.8	0.56	3.5	0.19	0.53
Lateral lunge trunk flexion, left value (°)	45.9 ± 10.2	46.4 ± 10.0	0.86	0.6	0.06	0.97
Lateral lunge trunk flexion, right value (°)	46.7 ± 9.6	47.1 ± 9.0	0.88	0.5	0.05	0.98
Lateral lunge pelvic obliquity, left value (°)	5.4 ± 10.8	4.7 ± 10.9	0.83	0.7	0.06	0.70
Lateral lunge pelvic obliquity, right value (°)	9.6 ± 8.2	5.4 ± 10.0	0.16	4.1	0.45	0.38
Lateral lunge hip adduction, left value (°)	-15.1 ± 11.5	-16.8 ± 11.1	0.64	1.7	0.15	0.94
Lateral lunge hip adduction, right value (°)	-16.4 ± 13.6	-12.7 ± 10.4	0.34	3.7	0.31	0.76
Lateral lunge dynamic valgus, left value (°)	6.9 ± 4.5	7.4 ± 4.13	0.74	0.5	0.14	0.73
Lateral lunge dynamic valgus, right value (°)	7.4 ± 2.6	7.0 ± 2.5	0.64	0.4	0.15	0.51
Vertical Jump						
Vertical jump center of mass height (cm)	19.3 ± 10.9	18.9 ± 10.5	0.90	0.4	0.04	0.98
Vertical jump eccentric phase hip flexion, maximum left value (°)	66.1 ± 19.5	70.4 ± 21.3	0.51	4.3	0.21	0.82
Vertical jump eccentric phase hip flexion, maximum right value (°)	62.6 ± 17.1	68.7 ± 19.8	0.30	6.1	0.33	0.73
Vertical jump eccentric phase knee flexion, maximum left value (°)	99.0 ± 17.6	97.3 ± 15.9	0.74	1.8	0.10	0.94
Vertical jump eccentric phase knee flexion, maximum right value (°)	100.6 ± 18.4	96.6 ± 17.0	0.48	4.0	0.23	0.91

Vertical jump eccentric phase ankle flexion, maximum left value (°)	40.8 ± 12.2	38.6 ± 7.3	0.51	2.1	0.21	0.52
Vertical jump eccentric phase ankle flexion, maximum right value (°)	37.2 ± 13.1	32.7 ± 7.6	0.19	4.5	0.42	0.64
Vertical jump eccentric phase lower extremity flexion, sum left value (°)†	207.2	207.9	0.90	1.1	0.04	0.93
Vertical jump eccentric phase lower extremity flexion, sum right value (°)†	205.7 ± 32.2	204.2 ± 29.9	0.88	1.5	0.05	0.93
Vertical jump center of mass eccentric depth (cm) †	19.3	18.9	0.93	0.6	0.03	0.94
Vertical jump eccentric phase dynamic valgus, left value (°)	9.9 ± 6.0	6.0 ± 4.3	0.02*	3.9	0.75	0.35
Vertical jump eccentric phase dynamic valgus, right value (°)	8.0 ± 4.1	10.0 ± 4.6	0.17	1.9	0.44	0.33
Vertical jump landing hip flexion, maximum left value (°)	37.1 ± 26.3	47.4 ± 25.8	0.22	10.2	0.39	0.82
Vertical jump landing hip flexion, maximum right value (°)	34.5 ± 25.5	45.9 ± 24.0	0.15	11.4	0.46	0.63
Vertical jump landing knee flexion, maximum left value (°)	82.5 ± 23.2	80.7 ± 22.0	0.80	1.8	0.08	0.97
Vertical jump landing knee flexion, maximum right value (°)	82.2 ± 22.5	79.1 ± 20.9	0.66	3.0	0.14	0.97
Vertical jump landing ankle flexion, maximum left value (°)	36.3 ± 10.2	35.3 ± 8.6	0.73	1.0	0.11	0.83
Vertical jump landing ankle flexion, maximum right value (°)	39.1 ± 8.7	35.8 ± 8.2	0.23	3.3	0.39	0.77
Vertical jump landing lower extremity flexion, sum left value (°)	152.3 ± 49.2	160.4 ± 46.4	0.60	8.1	0.17	0.93
Vertical jump landing lower extremity flexion, sum right value (°)	152.7 ± 44.0	157.9 ± 41.4	0.70	5.2	0.12	0.90
Vertical jump landing dynamic valgus, left value (°)	2.3 ± 5.8	-1.3 ± 5.8	0.06	3.6	0.62	0.34
Vertical jump landing dynamic valgus, right value (°)	4.4 ± 7.9	5.0 ± 8.6	0.82	0.6	0.07	0.75
Drop Jump						
Drop jump landing ankle flexion, left value (°)†	44.7	40.6	0.76	1.1	0.10	0.29
Drop jump landing ankle flexion, right value (°)†	45.0	40.1	0.28	2.9	0.36	0.39
Drop jump landing knee flexion, left value (°)	103.6 ± 15.6	100.9 ± 15.0	0.59	2.7	0.17	0.90
Drop jump landing knee flexion, right value (°)	102.3 ± 15.0	99.8 ± 15.0	0.61	2.5	0.16	0.90
Drop jump landing hip flexion, left value (°)	44.2 ± 27.0	47.8 ± 27.2	0.67	3.7	0.14	0.93
Drop jump landing hip flexion, right value (°)	39.9 ± 26.0	46.9 ± 25.2	0.39	7.0	0.27	0.88
Drop jump landing lower extremity flexion, sum left value (°)	185.6 ± 53.5	190.6 ± 41.8	0.75	5.0	0.10	0.87
Drop jump landing lower extremity flexion, sum right value (°)	187.4 ± 38.9	189.3 ± 40.9	0.88	1.9	0.05	0.97
Drop jump landing stiffness COM depth (cm)	29.6 ± 8.7	29.1 ± 8.4	0.84	0.6	0.07	0.95
Drop jump landing dynamic valgus, left value (°)	8.9 ± 11.3	2.81 ± 7.8	0.06	6.1	0.62	0.29

Drop jump landing dynamic valgus, right value (°)	5.6 ± 10.0	12.3 ± 13.3	0.08	6.7	0.57	0.49
Static Vertical Jump (concentric only)						
Static VJ center of mass height (cm)						
Static VJ hip flexion, maximum left value (°)	66.1 ± 19.5	70.4 ± 21.3	0.51	4.3	0.21	0.82
Static VJ hip flexion, maximum right value (°)	62.6 ± 17.1	68.7 ± 19.8	0.30	6.1	0.33	0.73
Static VJ knee flexion, maximum left value (°)	99.0 ± 17.6	97.3 ± 15.9	0.74	1.8	0.10	0.94
Static VJ knee flexion, maximum right value (°)	100.6 ± 18.4	96.6 ± 17.0	0.48	4.0	0.23	0.91
Static VJ ankle flexion, maximum left value (°)†	32.2	31.1	0.48	2.1	0.23	0.37
Static VJ ankle flexion, maximum right value (°)	37.2 ± 13.1	32.7 ± 7.6	0.19	4.5	0.42	0.64
Static VJ lower extremity flexion, sum left value (°)	198.0 ± 41.3	199.2 ± 34.1	0.92	1.2	0.03	0.91
Static VJ lower extremity flexion, sum right value (°)	200.4 ± 40.3	198.0 ± 35.3	0.84	2.4	0.06	0.95
Static VJ center of mass eccentric depth (cm)†	15.9	15.5	0.88	0.2	0.05	0.98
Static VJ dynamic valgus, left value (°)	8.0 ± 5.6	6.1 ± 4.8	0.24	2.0	0.38	0.75
Static VJ dynamic valgus, right value (°)	8.9 ± 6.3	9.5 ± 5.9	0.78	0.6	0.10	0.74
Static VJ landing hip flexion, maximum left value (°)	37.1 ± 26.3	47.4 ±25.8	0.22	10.2	0.39	0.82
Static VJ landing hip flexion, maximum right value (°)	34.5 ± 25.5	45.9 ± 24.0	0.15	11.4	0.46	0.63
Static Vertical Jump (concentric only)						
Static VJ landing knee flexion, maximum left value (°)†	77.9	74.1	0.86	0.7	0.06	0.97
Static VJ landing knee flexion, maximum right value (°)	79.1 ± 19.5	76.3 ± 16.9	0.62	2.9	0.16	0.94
Static VJ landing ankle flexion, maximum left value (°)	36.3 ± 10.2	35.3 ± 8.6	0.73	1.0	0.11	0.83
Static VJ landing ankle flexion, maximum right value (°)	39.1 ± 8.7	35.8 ± 8.2	0.23	3.3	0.39	0.77
Static VJ landing dynamic valgus, left value (°)	2.4 ± 9.3	0.8 ± 7.7	0.55	1.6	0.19	0.75
Static VJ landing dynamic valgus, right value (°)	2.9 ± 6.7	5.8 ± 8.5	0.44	1.9	0.25	0.74
Static VJ landing lower extremity flexion, sum left value (°)	152.3 ± 49.2	160.4 ± 46.4	0.60	8.1	0.17	0.93
Static VJ landing lower extremity flexion, sum right value (°)	152.7 ± 44.0	157.9 ± 41.4	0.70	5.2	0.12	0.90
Unilateral Vertical Jump						
Unilateral VJ center of mass height, left value (cm)	9.6 ± 7.5	9.5 ± 7.0	0.97	0.1	0.01	0.96
Unilateral VJ center of mass height, right value (cm)	9.4 ± 4.7	9.5 ± 4.2	0.94	0.1	0.02	0.93

Unilateral VJ hip flexion, maximum left value (°)	47.5 ± 26.3	51.8 ± 22.6	0.58	4.4	0.18	0.91
Unilateral VJ hip flexion, maximum right value (°)†	51.6	55.7	0.33	5.5	0.32	0.83
Unilateral VJ knee flexion, maximum left value (°)†	77.3	79.9	0.58	1.8	0.18	0.91
Unilateral VJ knee flexion, maximum right value (°)	82.3 ± 12.1	81.7 ± 10.8	0.88	0.6	0.05	0.92
Unilateral VJ ankle flexion, maximum left value (°)†	31.9	34.4	0.33	2.4	0.32	0.60
Unilateral VJ ankle flexion, maximum right value (°)	32.0 ± 7.5	33.2 ± 5.8	0.59	1.2	0.17	0.54
Unilateral VJ lower extremity flexion, sum left value (°)	158.6 ± 46.8	167.6 ± 36.5	0.50	9.0	0.21	0.92
Unilateral VJ lower extremity flexion, sum right value (°)	159.6 ± 32.1	166.4 ± 30.5	0.50	6.8	0.22	0.93
Unilateral VJ dynamic valgus, left value (°)	21.3 ± 12.8	18.3 ± 7.6	0.37	3.0	0.29	0.50
Unilateral VJ dynamic valgus, right value (°)	23.0 ± 11.7	26.8 ± 9.7	0.28	3.8	0.35	0.79
Unilateral VJ landing hip flexion, maximum left value (°)†	25.9	31.1	0.25	5.6	0.37	0.72
Unilateral VJ landing hip flexion, maximum right value (°)	35.1 ± 16.0	36.8 ± 15.3	0.74	1.7	0.11	0.82
Unilateral VJ landing knee flexion, maximum left value (°)	63.3 ± 16.3	62.7 ± 16.5	0.91	0.6	0.04	0.97
Unilateral VJ landing knee flexion, maximum right value (°)†	67.7	65.9	0.90	0.4	0.04	0.96
Unilateral Vertical Jump						
Unilateral VJ landing ankle flexion, maximum left value (°)†	34.3	36.9	0.57	1.2	0.19	0.70
Unilateral VJ landing ankle flexion, maximum right value (°)	31.0 ± 10.5	33.3 ± 7.3	0.43	2.3	0.25	0.71
Unilateral VJ landing lower extremity flexion, sum left value (°)	124.9 ± 40.3	134.1 ± 39.5	0.47	9.2	0.23	0.93
Unilateral VJ landing lower extremity flexion, sum right value (°)†	141.0	141.6	0.58	4.9	0.18	0.91
Unilateral VJ landing dynamic valgus, left value (°)	8.4 ± 9.3	7.9 ± 8.3	0.87	0.5	0.05	0.58
Unilateral VJ landing dynamic valgus, right value (°)	10.2 ± 8.2	11.8		0.53	1.6	0.20
Lateral Bound						
Lateral bound landing ankle flexion, left value (°)†	28.6	30.7	0.46	1.6	0.24	0.60
Lateral bound landing ankle flexion, right value (°)†	30.2	27.9	0.30	2.3	0.34	0.54
Lateral bound landing knee flexion, left value (°)	69.6 ± 12.6	69.3 ± 12.6	0.93	0.4	0.03	0.94
Lateral bound landing knee flexion, right value (°)†	68.6	69.5	0.55	2.4	0.20	0.74
Lateral bound landing hip flexion, left value (°)	43.3 ± 25.8	50.6 ± 26.2	0.38	7.4	0.28	0.84
Lateral bound landing hip flexion, right value (°)	42.4 ± 22.6	51.2 ± 23.9	0.24	8.9	0.38	0.61

Lateral bound landing lower extremity, sum left value (°)	140.5 ± 40.9	150.6 ± 38.1	0.42	10.1	0.26	0.89
Lateral bound landing lower extremity, sum right value (°)†	155.4	155.5	0.68	3.4	0.14	0.77
Lateral bound landing center of mass depth, left value (cm)†	13.3	12.9	0.92	0.2	0.03	0.96
Lateral bound landing center of mass depth, right value (cm)†	14.2	14.0	0.90	0.2	0.04	0.85
Multi-Hop						
Multi-hop eccentric phase hip flexion, maximum left value (°)†	11.9	17.2	0.17	5.8	0.45	0.79
Multi-hop eccentric phase hip flexion, maximum right value (°)	10.8 ± 13.7	14.2 ± 13.4	0.43	3.4	0.25	0.85
Multi-hop eccentric phase knee flexion, maximum left value (°)	54.0 ± 9.9	50.8 ± 9.2	0.31	3.2	0.33	0.88
Multi-hop eccentric phase knee flexion, maximum right value (°)	54.0 ± 9.6	50.7 ± 9.4	0.27	3.4	0.35	0.85
Multi-hop eccentric phase ankle flexion, maximum left value (°)†	33.6	36.9	0.51	1.6	0.22	0.69
Multi-hop eccentric phase ankle flexion, maximum right value (°)†	31.6	34.8	0.48	1.4	0.23	0.77
Multi-Hop						
Multi-hop eccentric phase lower extremity flexion, sum left value (°)	96.6 ± 26.6	101.0 ± 29.2	0.62	4.4	0.16	0.85
Multi-hop eccentric phase lower extremity flexion, sum right value (°)	96.0 ± 26.2	98.4 ± 23.8	0.77	2.3	0.09	0.92
Multi-hop stiffness depth, left value (°)	6.7 ± 4.2	6.3 ± 3.2	0.74	0.4	0.10	0.91
Multi-hop stiffness depth, right value (°)†	6.8	6.2	0.52	0.6	0.21	0.90

*Note: COM – center of mass; VJ – vertical jump; † not normally distributed, non-parametric statistical analysis; * p<0.05.*
